# Retinal Vasculometry Associations With Glaucoma: Findings From the European Prospective Investigation of Cancer–Norfolk Eye Study

**DOI:** 10.1016/j.ajo.2020.07.027

**Published:** 2020-12

**Authors:** Alicja R. Rudnicka, Christopher G. Owen, Roshan A. Welikala, Sarah A. Barman, Peter H. Whincup, David P. Strachan, Michelle P.Y. Chan, Anthony P. Khawaja, David C. Broadway, Robert Luben, Shabina A. Hayat, Kay-Tee Khaw, Paul J. Foster

**Affiliations:** a Population Health Research Institute, St George's, University of London, London, United Kingdom; b Faculty of Science, Engineering and Computing, Kingston University, Surrey, United Kingdom; c NIHR Biomedical Research Centre at Moorfields Eye Hospital and UCL Institute of Ophthalmology, London, United Kingdom; d Norfolk & Norwich University Hospital and University of East Anglia, Norwich, United Kingdom; e Department of Public Health and Primary Care, Institute of Public Health, University of Cambridge, Cambridge, United Kingdom; f Integrative Epidemiology Research Group, UCL Institute of Ophthalmology, London, United Kingdom

## Abstract

**Purpose:**

To examine retinal vasculometry associations with different glaucomas in older British people.

**Design:**

Cross-sectional study.

**Methods:**

A total of 8,623 European Prospective Investigation into Cancer-Norfolk Eye study participants were examined, who underwent retinal imaging, ocular biometry assessment, and clinical ascertainment of ocular hypertensive or glaucoma status (including glaucoma suspect [GS], high-tension open-angle glaucoma [HTG], and normal-tension glaucoma [NTG]). Automated measures of arteriolar and venular tortuosity, area, and width from retinal images were obtained. MainOutcomeMeasures: Associations between glaucoma and retinal vasculometry outcomes were analyzed using multilevel linear regression, adjusted for age, sex, height, axial length, intraocular and systemic blood pressure, and within-person clustering, to provide absolute differences in width and area, and percentage differences in vessel tortuosity. Presence or absence of within-person-between-eye differences in retinal vasculometry by diagnoses were examined.

**Results:**

A total of 565,593 vessel segments from 5,947 participants (mean age 67.6 years, SD 7.6 years, 57% women) were included; numbers with HTG, NTG, and GS in at least 1 eye were 87, 82, and 439, respectively. Thinner arterioles (−3.2 μm; 95% confidence interval [CI] −4.4 μm, −1.9 μm) and venules (−2.7 μm; 95% CI −4.9 μm, −0.5 μm) were associated with HTG. Reduced venular area was associated with HTG (−0.2 mm^2^; 95% CI −0.3 mm^2^, −0.1 mm^2^) and NTG (−0.2 mm^2^; 95% CI −0.3 mm^2^, −0.0 mm^2^). Less tortuous retinal arterioles and venules were associated with all glaucomas, but only significantly for GS (−3.9%; 95% CI −7.7%, −0.1% and −4.8%; 95% CI −7.4%, −2.1%, respectively). There was no evidence of within-person-between-eye differences in retinal vasculometry associations by diagnoses.

**Conclusions:**

Retinal vessel width associations with glaucoma and novel associations with vessel area and tortuosity, together with no evidence of within-person-between-eye differences in retinal vasculometry, suggest a vascular cause of glaucoma.

## Highlights

• Retinal vessel measurements, including (as a first report) vessel tortuosity and area, were associated with high-tension glaucoma and other glaucoma-related outcomes.
• Novel analyses showing that within-person-between-eye glaucoma diagnoses, intraocular pressure, and retinal vasculometry were uncorrelated provides further evidence that systemic microvascular changes may cause glaucoma.

Glaucoma is the leading global cause of irreversible visual impairment^1^ and a common cause of registered blindness.^2^ Glaucoma includes a heterogeneous group of diseases that result in optic neuropathy and progressive retinal ganglion cell degeneration, leading to visual loss.^3^ Primary open-angle glaucoma (POAG) is the most common type of glaucoma, accounting for three-quarters (74%) of all glaucoma cases.^4^ A recent review estimated the global number of POAG cases in 2020 to be 66 million, and predicted to rise owing to population aging.^5^ Elevated intraocular pressure (IOP) is the major modifiable risk factor for glaucoma. Pharmaceutical and/or surgical intervention to reduce IOP offers the accepted and only proven form of management.^6^, ^7^, ^8^ However, these management strategies to reduce IOP are not universally effective. Hence the exact etiology and therapeutic target for glaucoma remains unclear. Retinal vasculometry associations with glaucoma^9^ and associations of glaucoma with other vascular-related outcomes, including diabetes and cardiovascular events, suggests a vasculogenesis.^10^, ^11^, ^12^ However, it is unclear whether retinal vascular changes (particularly retinal arteriolar thinning)^13^^,^^14^ are a cause or consequence of glaucomatous retinal nerve fiber layer (RNFL) atrophy, particularly as evidence from longitudinal studies has been mixed.^15^^,^^16^ Moreover, whether other morphometric vascular changes beyond vessel thinning (including novel measures of vessel tortuosity and area, which have been little studied to date), of arterioles, venules, or both,^9^^,^^13^^,^^14^^,^^17^ are equally or differently indicative of the disease.^18^

We used a fully automated retina vasculometry system (QUARTZ) to examine associations with glaucoma in a large population of older British men and women, who took part in the European Prospective Investigation into Cancer (EPIC) Norfolk Eye study.^19^ The study allowed associations previously observed with retinal vessel width to be confirmed and to also examine novel associations with measures of vessel tortuosity and vessel area, providing the opportunity to further characterize the epiphenomenon of retinal vessel changes associated with glaucoma. Because the retinal vessels do not supply the anterior chamber of the eye, an association between retinal vascular morphology and glaucomatous disease could arise either as a consequence of raised IOP or because the retinal vessels reflect systemic changes in the microvasculature that are part of the causal pathway leading to glaucomatous retinal ganglion cell death independently of IOP. In this paper we also present an innovative analytic approach of examining within-person-between-eye correlations between IOP and retinal vascular morphology to provide further evidence (in the absence of consistent evidence from longitudinal studies) of whether retinal changes are a cause or consequence of glaucomatous disease.

## Methods

### Study Population

The EPIC study is a pan-European cohort study designed to investigate the causes of major chronic diseases.^20^ EPIC-Norfolk was the UK component of the study, and at baseline (from 1993 to 1997) recruited 25,639 participants (99.7% white European, aged 40-79 years) from 35 general practices in and around the city of Norfolk.^21^^,^^22^ Study participants had a detailed examination (including anthropometry, blood pressure, and urine and venous blood sampling) and questionnaire assessment at entry (including information on pre-existing cardiovascular disease, type 2 diabetes, and other medical conditions) and completed periodic questionnaires about their health (with a particular focus on dietary habits and smoking status). Participants underwent multiple clinical assessments, including repeat anthropometric assessment, venous blood sampling, retinal imaging (the EPIC Norfolk Eye Study) and physiological measures.^22^

### EPIC Norfolk Eye Study

Between 2004 and 2011 at the third clinical follow-up assessment, 8,623 participants provided updated information on medical history and lifestyle behavior.^23^ The study was carried out following the principles of the Declaration of Helsinki and the Research Governance Framework for Health and Social Care. The study was approved by the Norfolk Local Research Ethics Committee (05/Q0101/191) and East Norfolk and Waveney NHS Research Governance Committee (2005EC07L). All participants gave written, informed consent. Weight and height were measured with participants in light clothing without shoes. Weight was measured to the nearest 0.1 kg using regularly calibrated digital scales (Tanita TBF-300; Tanita UK Ltd, Middlesex, UK) and height to the last complete 0.1 cm using a stadiometer (Chasmors, London, UK). Body mass index (BMI) was calculated as weight/height squared in kg/m^2^. Seated blood pressure was measured twice using an automated blood pressure monitor (Accutorr Plus; Datascope Patient Monitoring, Huntington, UK); the mean of both measures was used. A nonfasting venous blood sample was collected; details of the analytic measures have been published previously.^22^ HbA1c was measured in whole blood using high-performance liquid chromatography. Serum total cholesterol and HDL-cholesterol were measured using an auto-analyzer (RA 1000 Technicon; Bayer Diagnostics, Basingstoke, UK); LDL-cholesterol was calculated using the Fredrickson–Friedewald equation.^24^

### Ocular Assessment

Ophthalmic tests included measurement of vision, visual acuity (logMAR acuity), and closed-field autorefraction (Humphrey model 500; Humphrey Instruments, San Leandro, California, USA), which was used to estimate axial length. Noncontact tonometry (using the AT555 and Ocular Response Analyzer; Reichert Corporation, Philadelphia, Pennsylvania, USA) provided gold-standard Goldmann-correlated IOP (mm Hg), as well as a measure of corneal hysteresis (to provide a corneal-compensated IOP).^25^ Scanning laser ophthalmoscopy of the optic nerve head (Heidelberg Retina Tomograph–HRT II, Heidelberg Engineering, Heidelberg, Germany) and polarimetry of the peripapillary nerve fiber layer (GDx VCC; Zeiss, Dublin, California, USA) were used to assess glaucomatous status. A 24-2 central threshold visual field test (Humphrey 750i Visual Field Analyzer; Carl Zeiss Meditec, Welwyn Garden City, UK) was carried out in those with abnormal imaging and in 10% of those with normal findings.

### Glaucoma Diagnosis

Glaucoma was defined as having structural abnormalities of the optic disc and visual field loss, in the absence of any other explanation.^26^ Higher-tension POAG and normal-tension glaucoma (NTG) were differentiated by pretreatment IOP (where median IOP >22 mm Hg determined high-tension glaucoma [HTG]). Glaucoma suspect (GS) was defined as the presence of early or minor glaucomatous disc features, associated with a normal visual field or the absence of visual field data. Those with IOP >21 mm Hg without visual field loss or optic disc abnormality were defined as ocular hypertensive (OHT). Specific quantitative methods and principles for diagnosis of glaucoma, GS, and OHT status followed the diagnostic principles from the International Society of Geographical and Epidemiological Ophthalmology.^26^ To limit false-positive or false-negative results, another consultant glaucoma ophthalmologist (P.J.F.) reviewed all examination findings and history in a subset of high-risk participants.^27^

### Fundus Imaging and Image Processing

Macular-centered 45-degree digital fundus photographs were taken using a TRC-NW6S nonmydriatic retinal camera and IMAGEnet Telemedicine System (Topcon Corporation, Tokyo, Japan) with a 10 megapixel Nikon D80 camera (Nikon Corporation, Tokyo, Japan) without pharmacologic dilation of the pupil. Image processing was carried out using an automated computerized vasculometry system (Quantitative analysis of retinal vessel topology and size; QUARTZ).^28^, ^29^, ^30^, ^31^ The QUARTZ system obtained thousands of measures of width and tortuosity from the whole retinal image (dependent on image quality), not just concentric areas centered on the disc (Supplemental Figure 1; Supplemental Material available at AJO.com). The QUARTZ measures were summarized using mean vessel segment width in micrometers and tortuosity with arbitrary units,^19^ weighted by the length of the vessel segment, for arterioles and venules separately for each image; mean segment widths and lengths were summed to calculate arteriolar and venular area in mm.^2^ A previously validated tortuosity measure that shows good agreement with subjective assessment of vessel tortuosity, based on the mean change in chord length between successive divisions of the vessel, was used.^19^ Automated image quality assessment allowed the best image per individual to be used. System performance had been validated previously and allowed fully automated batch processing of images from large population-based studies.^28^, ^29^, ^30^, ^31^ A model eye was used to quantify the magnification characteristics of the imaging system used, allowing pixel dimensions of vessel width to be converted to real size.^32^

### Statistical Analysis

Statistical analyses were carried out using STATA software (version 15; StataCorp LP, College Station, Texas, USA). Histograms of retinal vessel widths and area showed normal distributions, while measures of tortuosity were positively skewed and log-transformed. Multilevel linear regression models adjusting for age and sex were used to examine associations of prevalent glaucoma status for each eye in relation to retinal vessel measures for the corresponding eye. Eye-level as opposed to person-level glaucoma diagnoses were considered a priori to assess local vasculometry associations, to allow for between-eye differences in glaucoma diagnoses within the same individual. A random effect for person was included in all regression models to allow for the right- and left-eye data from the same person to contribute to the analyses. Regression models provided mean differences in width (μm) and area (mm^2^) and percentage differences in tortuosity (as log transformed) for venules and arterioles separately for each type of glaucoma compared with those without a diagnosis of glaucoma. Models were adjusted for (1) age and sex (Model 1), followed by additional adjustment for (2) stature (ie, height), ocular biometry (ie, axial length), and factors related to ocular hemodynamics (corneal-compensated IOP and systolic blood pressure; Model 2); and (3) smoking status (never, former, current) and cardiometabolic risk factors (including total cholesterol, HbA1c, and BMI; Model 3). Model 2 was used as the primary association, given independence from IOP and blood pressure, which show strong associations with retinal vasculometry.^33^ Sensitivity analyses examined the effect of excluding participants with self-reported heart attack, stroke, type 2 diabetes, and hypertension.

#### Between-Person Analyses

Among individuals with retinal images from both eyes and with the same diagnosis in each eye, we examined cross-sectional associations between vasculometric measures and IOP to assess the consistency of this relationship among those with and without a glaucoma diagnosis.

#### Within-Person-Between-Eye Analyses

**Figure 1:**
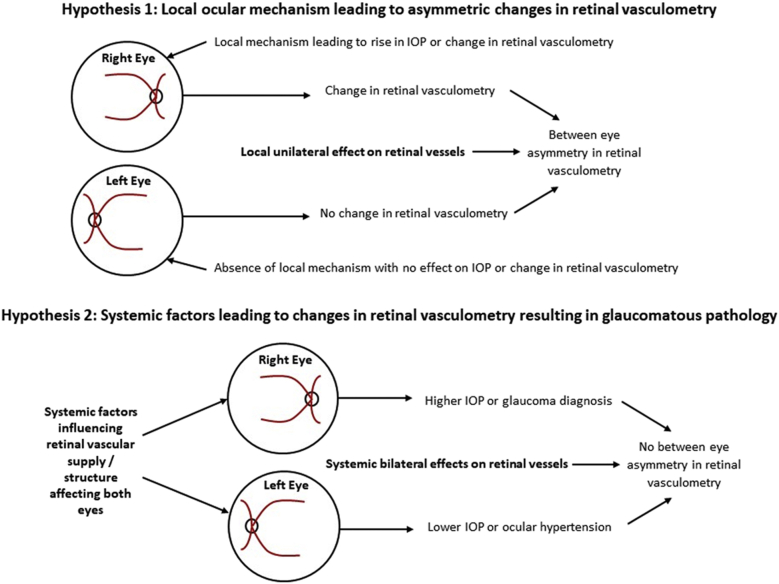
Pathways by which local and systemic factors may influence retinal vasculometry comparisons between eyes.

We examined within-person-between-eye differences in retinal vessel measures in relation to within-person-between-eye differences in IOP and specifically among individuals with a different diagnosis between eyes. Although IOP is a modifiable risk factor for glaucoma, it is not a defining feature, akin to high blood pressure being a modifiable risk factor for coronary heart disease. However, within-person-between-eye asymmetry in IOP generally relates to severity of glaucoma, in that the eye with the higher IOP usually shows more advanced disease.^34^, ^35^, ^36^ Hence, at the person level asymmetry in IOP could be used as a marker of asymmetry in disease severity (rather than a defining feature of the disease itself). Similarly, within-person-between-eye asymmetry in glaucoma diagnosis provides another within-person marker of asymmetry in disease type/severity. Within-person-between-eye asymmetry in IOP or glaucoma diagnosis could then be used to explore within-person-between-eye asymmetry in retinal vasculometry. A major advantage of this approach is that within-person-between-eye analyses would be self-controlled for systemic cardiovascular risk factors, lifestyle, medications and other unmeasured confounders. If within-person-between-eye asymmetry glaucoma severity is in part due to a local ocular pathway, then within-person-between-eye asymmetry in retinal vasculometry might occur as a consequence of local ocular pathogenic mechanisms. However, if the effect on the retinal vasculometry is not influenced by local ocular mechanisms but instead is influenced by factors higher upstream (ie, systemic factors), then within-person-between-eye asymmetry in retinal vasculometry would not be observed. Hence, absence of an association of within-person-between-eye differences in IOP or glaucoma diagnosis with within-person-between-eye differences in retinal vasculometry would suggest that any differences in vessel characteristics are unlikely to be a consequence of the disease process occurring within the eye but potentially a precursor, on the causal pathway, or indicative of other systemic causes. However, limitations of sample size need to be acknowledged, in that there may be insufficient power to detect within-person-between-eye differences in association and large studies are needed to mitigate against this. These 2 hypotheses are illustrated by pathway diagrams in Figure 1. As far as we are aware, we are the first to use within-person-between-eye differences in retinal vasculometry by glaucoma diagnosis, to provide further evidence (but not absolute proof) of whether retinal vessel changes are a cause or consequence of glaucomatous disease.

## Results

In total 8,623 individuals participated in the EPIC Norfolk Eye Study (mean age 68 years, 57% women), of 18,380 invited (participation rate 47%). Characteristics of EPIC Norfolk participants who took part in the Eye Study with and without usable fundus images have been described previously.^23^ Those taking part were younger at baseline, were of higher BMI and socioeconomic status, and were less likely to be a current smoker compared to participants not followed up.^23^ Of the 7,411 individuals who underwent fundus imaging and refractive assessment, 5,947 (80%) had a fundus image from at least 1 eye of sufficient quality for analysis. Images excluded were mis-centered, defocused, or obstructed by lashes and/or media opacities. Participant characteristics of the 5,947 included and 2,676 excluded from the vasculometry analyses are summarized in Table 1. Characteristics were similar, except those included were younger (67.6 years vs 71.3 years) and more likely to be women (57% vs 51%). The prevalences of HTG, NTG, GS, and OHT in at least 1 eye among the 5,947 participants included were 1.5% (n = 87), 1.4% (n = 82), 7.4%, (n = 439), and 10.8% (n = 642), respectively. In total, measures of vessel width and tortuosity for 565,593 vessel segments (279,802 arterioles, 285,791 venules) from 10,466 eyes were included in the analyses.

**Table 1:** Participant Characteristics of EPIC Study Participants who Took Part in the Third Health Check With and Without Useable Fundus Images

Characteristic	Included in the Analyses	Excluded from the Analyses
Total number	5,947	2,676
Age (y), mean (SD)	67.6 (7.6)	71.3 (8.6)
Sex, n (% female)	3,393 (57.1)	1,369 (51)
Current smoker, n (%)	267 (4.5)	105 (4)
Former smoker, n (%)	2,628 (44.2)	1,281 (48)
Height (cm), mean (SD)	166.4 (9.1)	166.2 (9.2)
Weight (kg), mean (SD)	74.4 (14.3)	74.6 (14.0)
BMI (kg/m^2^), mean (SD)	26.8 (4.3)	27.0 (4.2)
Systolic blood pressure (mm Hg), mean (SD)	135.7 (16.6)	137.2 (16.8)
Diastolic blood pressure (mm Hg), mean (SD)	78.4 (9.2)	77.9 (9.6)
Self-reported heart attack, n (%)	187 (3.1)	106 (4.0)
Self-reported stroke, n (%)	118 (2.0)	67 (2.5)
Self-reported type 2 diabetes, n (%)	237 (4.0)	156 (5.8)
Self-reported hypertension, n (%)	1,757 (29.5)	869 (32.5)
Ocular measures, mean (SD)		
Axial length OD (mm)	23.6 (1.2)	23.5 (1.2)
Axial length OS (mm)	23.5 (1.2)	23.5 (1.3)
Mean best vision sphere OD (D)	0.1 (2.2)	0.2 (2.5)
Mean best vision sphere OS (D)	0.2 (2.3)	0.3 (2.4)
IOPg OD (mm Hg)	16.1 (3.9)	
IOPg OS (mm Hg)	16.9 (3.9)	
IOPcc OD (mm Hg)	16.1 (3.8)	
IOPcc OS (mm Hg)	16.8 (3.9)	
Arteriolar width OD (μm)	74.9 (6.9)	
Arteriolar width OS (μm)	74.8 (6.7)	
Venular width OD (μm)	91.5 (11.3)	
Venular width OS (μm)	90.3 (11.9)	
Arteriolar area OD (mm^2^)	2.0 (0.8)	
Arteriolar area OS (mm^2^)	2.0 (0.8)	
Venular area OD (mm^2^)	2.6 (0.7)	
Venular area OS (mm^2^)	2.7 (0.7)	
Arteriolar tortuosity OD × 1,000^a^	4.2 (1.6)	
Arteriolar tortuosity OS × 1,000^a^	4.3 (1.6)	
Venular tortuosity right × 1,000^a^	3.1 (1.4)	
Venular tortuosity left × 1,000^a^	3.3 (1.4)	

D = diopters; GS = glaucoma suspect; HTG = high-tension open-angle glaucoma; IOPcc = corneal-compensated intraocular pressure; IOPg = Goldmann-correlated intraocular pressure; NTG = normal-tension glaucoma.

For participants included in the analyses best vision sphere missing for 27 participants.

a Geometric mean (GSD).

Eye-specific retinal vasculometry, IOP, and ocular biometry measures in those with usable fundus images are summarized in Table 1 for arterioles and venules separately, which overall appeared similar for right and left eyes. Mean arteriolar width was 74.9 μm (standard deviation [SD] 6.8 μm), venular width was 90.9 μm (SD 11.5 μm); corresponding measures of vessel area were 2.0 mm^2^ (SD 0.7 mm^2^) and 2.7 mm^2^ (SD 0.7 mm^2^), tortuosity 4.2 × 10^−^³ (GSD 1.6 × 10^−^³) and 3.2 × 10^−^³ (GSD 1.4 × 10^−^³), respectively, so arterioles were thinner (by 15 μm), less dense (by 26%), and more tortuous (by 40%) than venules.

**Figure 2:**
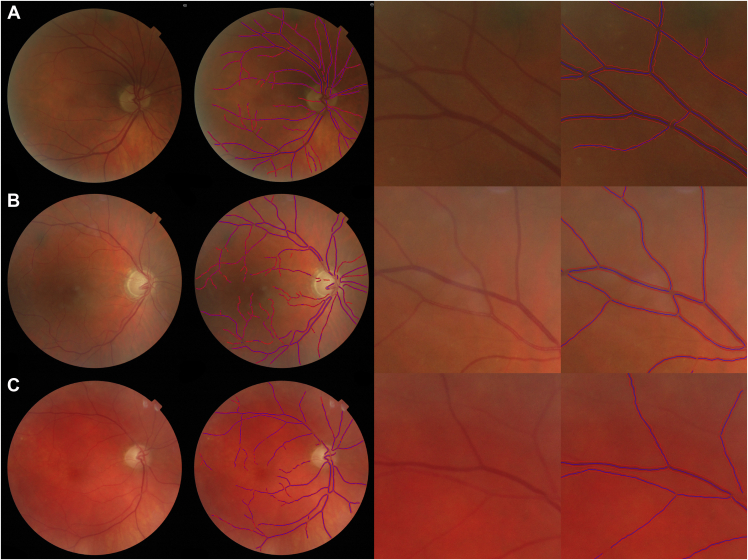
Demonstration of QUARTZ output showing narrow arterioles in the superior arcade of a patient with high-tension glaucoma (HTG) (B) and narrow venules in another patient with HTG (C), compared with a healthy subject (A).

Cross-sectional associations between eye-level retinal vessel measures and ocular diagnoses are shown in Table 2. Allowing for factors related to hemodynamics (IOP and systolic blood pressure) and biometry (axial length and height–Model 2), eyes with a diagnosis of HTG had on average narrower arterioles (by −3.2 μm, 95% confidence interval [CI] −4.4 μm, −1.9 μm) and narrower venules (−2.7 μm, 95% CI −4.9 μm, −0.5 μm; these effect sizes remained similar after adjustment for cardiometabolic risk factors (Model 3), perhaps becoming a little stronger (−3.8 μm, 95% CI −5.1 μm, −2.4 μm; −3.6 μm, 95% CI −6.0 μm, −1.3 μm, respectively). These associations are demonstrated visually in Figure 2, which shows narrower arterioles in the superior retinal vessel arcade of a patient with HTG (middle panel) and narrow venules in another patient with HTG (lower panel), compared with a healthy subject (upper panel).

**Table 2:** Adjusted Differences in Vessel Width and Tortuosity Associated With an Ocular Diagnosis of Glaucoma Compared With Those Without a Diagnosis of Glaucoma

Diagnosis	Absolute Difference in Arteriolar Width, μm (95% CI)						Absolute Difference in Venular Width, μm (95% CI)					
	Model 1	*P* Value	Model 2	*P* Value	Model 3	*P* Value	Model 1	*P* Value	Model 2	*P* Value	Model 3	*P* Value
HTG	−2.41 (−3.69, −1.12)	<.001	−3.17 (−4.43, −1.92)	<.001	−3.75 (−5.08, −2.41)	<.001	−2.67 (−4.82, −0.53)	.015	−2.70 (−4.89, −0.51)	.016	−3.64 (−5.95, −1.32)	.002
NTG	−1.22 (−2.54, 0.09)	.068	−2.12 (−3.41, −0.83)	.001	−1.75 (−3.10, −0.39)	.011	−1.03 (−3.23, 1.16)	.356	−1.06 (−3.29, 1.18)	.353	−1.25 (−3.60, 1.10)	.298
GS	0.36 (−0.22, 0.94)	.226	−0.22 (−0.80, 0.36)	.454	−0.22 (−0.83, 0.39)	.478	−0.24 (−1.21, 0.73)	.630	−0.38 (−1.39, 0.63)	.466	−0.61 (−1.67, 0.45)	.258
OHT	0.13 (−0.34, 0.60)	.585	−0.32 (−0.82, 0.18)	.212	−0.32 (−0.84, 0.21)	.240	0.13 (−0.66, 0.91)	.754	0.05 (−0.81, 0.91)	.913	−0.03 (−0.93, 0.87)	.948
	Absolute Difference in Arteriolar Area, mm^2^ (95% CI)						Absolute Difference in Venular Area, mm^2^ (95% CI)					
HTG	−0.04 (−0.18, 0.10)	.554	−0.07 (−0.21, 0.07)	.296	−0.09 (−0.24, 0.06)	.217	−0.10 (−0.23, 0.03)	.116	−0.21 (−0.34, −0.09)	.001	−0.16 (−0.29, −0.03)	.015
NTG	−0.09 (−0.23, 0.05)	.207	−0.09 (−0.23, 0.06)	.241	−0.10 (−0.25, 0.05)	.183	−0.05 (−0.18, 0.08)	.429	−0.15 (−0.28, −0.02)	.022	−0.12 (−0.25, 0.01)	.081
GS	−0.01 (−0.08, 0.05)	.650	−0.03 (−0.09, 0.04)	.370	−0.04 (−0.11, 0.02)	.198	0.04 (−0.02, 0.10)	.156	−0.02 (−0.08, 0.03)	.415	−0.03 (−0.09, 0.03)	.399
OHT	0.04 (−0.02, 0.09)	.170	0.00 (−0.06, 0.05)	.981	0.01 (−0.05, 0.07)	.696	0.01 (−0.03, 0.06)	.561	−0.03 (−0.08, 0.02)	.282	−0.02 (−0.07, 0.03)	.417
	% Difference in Arteriolar Tortuosity (95% CI)						% Difference in Venular Tortuosity (95% CI)					
HTG	−2.41 (−10.31, 6.19)	.571	−2.21 (−10.30, 6.60)	.611	−3.77 (−12.19, 5.44)	.410	−5.64 (−11.04, 0.09)	.053	−4.59 (−10.17, 1.34)	.126	−3.48 (−9.47, 2.91)	.279
NTG	−4.73 (−12.49, 3.72)	.264	−4.95 (−12.85, 3.66)	.251	−5.52 (−13.85, 3.61)	.227	−2.90 (−8.64, 3.19)	.342	−2.81 (−8.66, 3.42)	.369	−2.58 (−8.75, 4.01)	.434
GS	−2.80 (−6.44, 0.97)	.144	−3.94 (−7.67, −0.06)	.046	−4.77 (−8.67, −0.70)	.022	−4.83 (−7.35, −2.26)	<.001	−4.79 (−7.42, −2.10)	.001	−4.81 (−7.56, −1.98)	.001
OHT	−1.54 (−4.41, 1.43)	.306	−1.73 (−4.83, 1.46)	.284	−1.09 (−4.36, 2.29)	.522	−1.57 (−3.73, 0.64)	.162	−1.56 (−3.96, 0.89)	.211	−0.87 (−3.41, 1.73)	.507

GS = glaucoma suspect; HTG = high-tension open-angle glaucoma; NTG = normal-tension glaucoma; OHT = ocular hypertension.

Adjusted differences are from a multilevel model allowing for repeated images from the same person (random effect for person). Model 1 (N = 5,947) adjusted for age, sex as fixed effects; Model 2 = Model 1 + corneal-compensated intraocular pressure, systolic blood pressure, height, axial length; Model 3 = Model 2 + smoking status, total cholesterol, body mass index, and HbA1c. The sample size reduction due to missing data in Model 2 N = 5,742, Model 3 N = 5,204

NTG showed similar directions of association with vessel width, but were less in magnitude, only being statistically significant in Models 2 and 3 for arteriolar width (−2.1 μm, 95% CI −3.4 μm, −0.8 μm; −1.8 μm, 95% CI −3.1 μm, −0.4 μm, respectively). Reduced venular area was associated with both HTG (−0.2 mm^2^, 95% CI −0.3 mm^2^, −0.1 mm^2^) and NTG (−0.2 mm^2^, 95% CI −0.3 mm^2^, −0.0 mm^2^), which persisted after further adjustment for cardiometabolic risk factors (Model 3, −0.2 mm^2^, 95% CI −0.3 mm^2^, 0.0 mm^2^; −0.1 mm^2^, 95% CI −0.3 mm^2^, 0.0 mm^2^, respectively). While GS showed little association with venular and arteriolar width, arteriolar and venular tortuosity was reduced (−3.9%, 95% CI −7.7%, −0.1%; −4.8%, 95% CI −7.4%, −2.1%, respectively), even after adjustment for cardiometabolic risk factors (Model 3, −4.8%, 95% CI −8.7%, −0.7%; −4.8%, 95% CI −7.6%, −2.0%, respectively). Reduced arteriolar and venular tortuosity were also associated with HTG and NTG, but associations did not reach statistical significance at the 5% level. The associations between OHT and retinal vasculometry (both arteriolar and venular width and tortuosity) were null.

Sensitivity analyses, excluding those who self-reported heart attack, stroke, type 2 diabetes, and hypertension, resulted in fewer numbers (ie, 6,194 eyes from 3,516 participants) but showed similar directions of association, with HTG associations with arteriolar and venular width remaining (−3.8 μm, 95% CI −5.4 μm, −2.3 μm; −3.7 μm, 95% CI −6.4 μm, −1.0 μm, respectively), and decreased arteriolar and venular tortuosity with GS (−4.8%, 95% CI −9.5%, 0.2%; −4.1%, 95% CI −7.4%, −0.6%, respectively).

**Figure 3:**
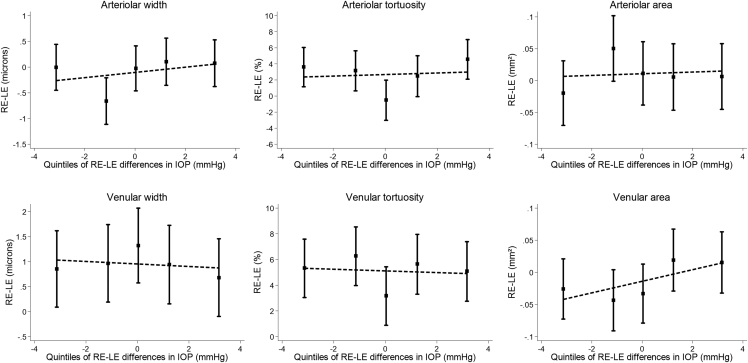
Average between-eye differences in retinal vessel measures by quintiles of between-eye differences in Goldmann-correlated intraocular pressure (IOP) among individuals who do not have a glaucoma diagnosis in either eye. R2 values from linear regression using IOP as a continuous variable are less than 0.001 in all instances.

Among individuals with the same diagnosis in each eye, arteriolar width marginally increased positively with IOP. A similar pattern of association was observed among those unaffected by a glaucoma in either eye, GS in both eyes, or OHT in both eyes. However, the pattern was less clear among NTG and HTG patients, as the 95% CIs are wide with small numbers of cases (Supplemental Figure 2; Supplemental Material available at AJO.com). Table 3 summarizes the between-eye asymmetry in IOP and retinal vasculometry. Despite there being some evidence of asymmetry between the eyes, there was no evidence to suggest that asymmetry in retinal vasculometry related to between-eye differences in either Goldmann-correlated IOP (Figure 3) or corneal-compensated IOP (Supplemental Figure 3; Supplemental Material available at AJO.com).

**Table 3:** Between-Eye Differences in Intraocular Pressure and Retinal Vessel Measures Among Individuals With the Same Diagnosis in Each Eye

Both Eyes Classified as	N	Average Between-Eye Differences, (SD) [95% CI]							
		IOPg	IOPcc	Vessel Width (μm)		Vessel Tortuosity (%)		Vessel Area (mm^2^)	
				Arteriolar	Venular	Arteriolar	Venular	Arteriolar	Venular
Unaffected	3,408	0.0 (2.3)	−0.1 (2.9)	−0.1 (6.0)	0.9 (10.3)	2.6 (1.4)	5.0 (1.4)	0.01 (0.68)	−0.01 (0.63)
		[−0.1, 0.1]	[−0.2, 0.0]	[−0.3, 0.1]	[0.6, 1.3]	2.6 (1.4)	[6.0, 4.0]	[−0.01, 0.04]	[−0.04, 0.01]
HTG	32	0.1 (3.6)	0.0 (4.2)	−1.0 (7.9)	−1.6 (9.1)	−3.9 (1.5)	−6.8 (1.5)	0.00 (0.65)	0.07 (0.67)
		[−1.2, 1.4]	[−1.6, 1.5]	[−3.7, 1.8]	[−4.7, 1.6]	[9.0, −18.7]	[6.5, −22.0]	[−0.23, 0.23]	[−0.17, 0.31]
NTG	26	0.0 (2.9)	0.0 (3.4)	−1.4 (9.4)	0.8 (8.1)	5.9 (1.4)	11.5 (1.5)	−0.12 (0.68)	0.10 (0.76)
		[−1.2, 1.2]	[−1.4, 1.3]	[−5.1, 2.3]	[−2.4, 4.0]	[17.3, −7.0]	[23.6, −2.6]	[−0.39, 0.15]	[−0.20, 0.40]
GS	164	−0.1 (3.1)	−0.2 (3.4)	−0.3 (7.2)	−0.4 (11.1)	3.4 (1.4)	6.4 (1.4)	0.00 (0.71)	−0.06 (0.57)
		[−0.5, 0.4]	[−0.7, 0.4]	[−1.3, 0.8]	[−2.0, 1.3]	[8.0, −1.4]	[10.7, 1.9]	[−0.11, 0.10]	[−0.15, 0.02]
OHT	193	−0.5 (3.4)	−0.7 (4.1)	0.5 (6.6)	1.1 (10.8)	3.3 (1.4)	4.2 (1.4)	0.05 (0.70)	−0.01 (0.63)
		[−1.0, 0.0]	[−1.3, −0.1]	[−0.4, 1.4]	[−0.4, 2.6]	[7.9, −1.5]	[8.5, −0.2]	[−0.05, 0.15]	[−0.10, 0.07]

GS = glaucoma suspect; HTG = high-tension open-angle glaucoma; IOPcc = corneal-compensated intraocular pressure; IOPg = Goldmann-correlated intraocular pressure; N = number of participants; NTG = normal-tension glaucoma; OHT = ocular hypertension.

For tortuosity the SD if the exponent of geometric SD.

For participants with a different diagnosis between eyes, there was no clear evidence of systematic within-person-between-eye differences in retinal vasculometry (Supplemental Table; Supplemental Material available at AJO.com). The main subgroup pairs were as follows: unaffected in 1 eye and OHT in the other (n = 288); unaffected vs GS (n = 106); unaffected vs NTG (n = 10); NTG vs GS (n = 23); GS vs OHT (n = 31); HTG vs GS (n = 21). Furthermore, between-eye differences in Goldmann-correlated IOP were not related to between-eye differences in retinal vasculometry, except for arteriolar area (Supplemental Figure 4; Supplemental Material available at AJO.com).

## Discussion

In this large, extensively phenotyped predominantly White European cohort of middle-aged and older men and women, we showed that an ocular diagnosis of POAG (particularly HTG) was associated with reduced retinal arteriolar and venular width, and also with reduced venular area (which has been little studied to date). Smaller venular and arteriolar tortuosity were also associated with a diagnosis of GS, suggesting that retinal vessel tortuosity may be a cue to POAG development. The associations identified in the present study persisted with adjustment for factors related to ocular hemodynamics, for cardiovascular and metabolic risk factors, and after exclusion of those who self-reported cardiometabolic outcomes, including heart attack, stroke, type 2 diabetes, and hypertension.

The findings of reduced retinal arteriolar and venular width associated with POAG are in keeping with a number of population-based cross-sectional studies that have observed arteriolar,^9^^,^^13^ venular, or both arteriolar and venular narrowing associated with POAG.^14^^,^^17^ However, vessel width associations have not been observed in all studies.^37^ The orders of magnitude of arteriolar and venular thinning in this study (approximately 2-3 μm on average, with confidence intervals up to 6 μm) are commensurate with other studies, although some studies have shown mean differences in venular diameters of 10 μm or more, but in relation to much larger central retinal vein equivalent dimensions,^14^^,^^17^ which are double the magnitude of the direct measures of venular width observed in this study (ie, >200 μm vs <100 μm). Given these different methods of measurement, which have been previously shown to have poor agreement, effect sizes will inevitably vary, limiting direct comparisons across studies.^38^^,^^39^ Despite this, we believe our findings are similar proportionately and in absolute terms to other studies and are more likely to be statistically significant, given the large number of measures made (ie, with thousands of measures per eye). Moreover, our study used measures obtained over the entire retinal image, as opposed to measures restricted to peripapillary concentric areas around the optic disc,^9^^,^^13^^,^^14^^,^^17^^,^^37^ with dimensions similar to studies that have reported direct manual measures of peripapillary vessel widths,^13^ as opposed to extrapolating to central retinal artery and vein equivalents.^9^^,^^13^^,^^14^^,^^17^^,^^37^ Fewer population-based studies have reported vessel width associations with other glaucomatous outcomes, including GS and NTG.^9^^,^^17^ We found evidence of vessel thinning with NTG, although the magnitude of the difference was less than that observed with high-tension POAG (HTG) and only statistically significant for arterioles. Another study also showed vessel thinning with NTG, but this was only statistically significant for venules, suggesting that effect sizes might be smaller and that greater numbers are needed to provide evidence of effect.^17^

Taking our panretinal measurement approach, our findings suggest that glaucomatous vasculometric features associated with glaucoma are evident beyond previous, more restrictive peripaillary measurement zones, affecting both the macrovasculature and microvasculature and both arterioles and venules. Our findings are novel in also showing reduced venular area with glaucoma outcomes, and decreased vessel tortuosity with suspect glaucoma, which has been little studied to date. Reduced vessel tortuosity has been observed with POAG in 1 other study (in addition to other markers of vasculometry)^18^ as well as proxy glaucoma-related outcomes, including reduced neuroretinal rim area and RNFL thickness.^40^^,^^41^ An important feature of tortuosity is that, unlike absolute quantification of vessel width, measures were based on a ratio and hence were dimensionless, potentially being interpretable across different imaging systems. However, values are not directly comparable, since methods for calculating tortuosity differ and are not universal.

Another important consideration is whether vasculometry changes, and particularly novel associations observed with retinal vessel tortuosity, occur as a cause or consequence of glaucoma. The vasculogenesis of glaucoma is supported by a number of systemic observations, including glaucoma associations with increased tortuosity^42^ and reduced blood flow in the capillary nail bed,^43^ and associations observed with vasospastic conditions, such as migraine and Raynaud syndrome.^44^ However, biologically it is unclear whether RNFL changes associated with glaucoma result in vasoconstriction owing to reduced metabolic need or whether changes in markers of ocular perfusion/vasoautoregulation (including nitric oxide enzymes),^45^ leading to vascular dysregulation^46^^,^^47^ and/or aqueous oxidative stress, precede glaucomatous RNFL changes.^48^ We argue that the novel between-eye analysis included in the present study allows further examination of cause and consequence. Within-person correlations of vascular measures with IOP and/or glaucoma diagnosis would be self-controlled for systemic cardiovascular risk factors, lifestyle, medication, etc, and would therefore tend to argue in favor of consequence at the eye level, whereas the lack of within-person-between-eye correlation observed in the present study suggests that systemic microvascular changes, manifest in the retinal vessels but also affecting other parts of the eye, are a more likely explanation for the associations observed between glaucoma (or raised IOP) and retinal vascular morphology at the between-person level. In reality it is likely that the disease process is a combination of both systemic and local factors influencing the disease process. However, the analyses would indicate whether systemic factors might precede the disease process, in which case exploration of between-person-within-eye differences in retinal vasculometry prospectively with incident glaucoma cases would be needed to test this hypothesis. Unfortunately, longitudinal evidence from large population-based studies has been sparse and provided equivocal evidence, showing prospective associations between retinal vasculometry and subsequent glaucoma diagnosis,^15^ or no association at all.^16^ While within-person-between-eye analyses could be employed in other cross-sectional studies, especially given that such analyses are well powered, further evidence from longitudinal studies is needed to fully elucidate the causal sequence of events.

The present study had a number of strengths and weaknesses worthy of consideration. A major strength was the fully automated vasculometry system used (ie, QUARTZ), which has been extensively validated^28^, ^29^, ^30^, ^31^ and used to provide a detailed retinal vasculometry phenotype for this cohort,^19^ including novel characterization of vessel tortuosity and area. Fully automated approaches are key to large population study, where semiautomated/manual approaches are prohibitively labor intensive and costly. Although systemic markers may be risk factors for glaucoma, glaucoma is a localized disease state. Hence, the analytic approach was at an eye level, not at a person level, to allow for eye-specific diagnoses. Previous approaches have used a hierarchy to provide person-level diagnoses (even when there are diagnostic differences between eyes),^27^ which could potentially dilute local effects in this context. Accordingly, a person-based analysis using a hierarchy of diagnoses, that is, where (1) POAG (HTG), (2) GS, and (3) NTG are used in order of preference,^27^ showed similar but weaker vasculometry effects (data not presented). A major advantage of the EPIC-Norfolk cohort is that participants are extensively phenotyped, allowing vasculometry associations independent of potential confounders to be gauged. We chose to adjust for axial length to allow for the ocular magnification characteristics of participants, and stature, as this is inversely related to open angle glaucoma, as well as markers of ocular and systemic hemodynamics.^49^^,^^50^ This is why the primary analysis also adjusted for IOP and systolic blood pressure within this model, which allowed the independence of associations to be shown (particularly from blood pressure, where we have shown definitively strong inverse associations with these retinal vasculometric measures).^33^ The next model included additional adjustment for established cardiovascular and metabolic risk factors, including total cholesterol, HbA1c, BMI, and smoking status, to examine whether retinal vasculometry associations with glaucoma were independent of well-known precursors for cardiometabolic disease. This was confirmed in sensitivity analyses excluding participants who self-reported heart attack, stroke, type 2 diabetes, and hypertension (many of which have been proposed as putative risk factors for glaucoma), which further showed persistence of the retinal vasculometry-glaucoma associations observed. Moreover, we were able to fully account for any potential magnification effects of the imaging system used.^32^

In terms of weaknesses, the present study, although nested within a cohort, was cross-sectional, with the ocular assessment only being part of the third clinical examination^19^; hence it was not possible to establish the temporality of association. Also, the current study was opportunistic and not directly powered to detect within-person-between-eye differences in retinal vasculometry associations with glaucoma; hence a larger study might yield differences. Longitudinal studies are needed, which only future follow-up of the cohort would provide, and where there would be increased power to discern effects, with more events occurring owing to increased age.^5^ Moreover, it would also be possible to examine potential ocular and systemic antihypertensive treatment effects. Given the lack of longitudinal follow-up, we can also not rule out that the association of GS with arteriolar and venular tortuosity may reflect a diagnostic bias, where those with straighter vessels who may not go on to develop glaucoma are inadvertently labeled. While the observation of similar directions of vessel tortuosity associations with HTG and NTG would argue against this, follow-up of the cohort establishing those who convert to glaucoma is needed to formally address this issue further. There were also too few closed-angle glaucoma events to discern retinal vasculometry associations, which may reflect the extensively white European ancestry of the UK-based cohort, who are at lower risk. Moreover, the cohort, although generalizable to the UK, is somewhat select, which may infer a healthy volunteer bias.^27^

We have provided evidence that the width, area, and tortuosity of retinal vessels at a person level appear to be affected by glaucoma. Similar magnitudes and directions of association, particularly with vessel width, suggest that both arterioles and venules are affected, and that vascular effects are pervasive, not discriminatory. Moreover, the absence of between-eye-within-person correlations in retinal vasculometry by diagnoses would appear to rule out consequence, thereby suggesting that vasculometry changes are indicative of systemic causal factors, which could easily be confirmed in other cross-sectional studies. However, in the absence of further longitudinal data we are merely hypothesizing that our novel approach of showing a lack of within-person-between-eye differences in retinal vasculometry by glaucoma diagnosis in this cross-sectional study provides further evidence (but not absolute proof) of potential cause and effect. With the emergence of artificial intelligence approaches to detect glaucoma,^51^ vessel changes, in addition to other features, may be key areas of interest to discern presence or absence of disease, which can be more readily quantified compared with visual interpretation (Figure 2). Saliency maps may help in understanding the key features of the retinal image that determine presence of disease, as they have done in cardiovascular disease risk factor prediction.^52^ Such examination may reveal that retinal vessels are also key areas of interest in glaucomatous development and, given the limited value of IOP as a screening tool for glaucoma,^27^ vessel morphometry (particularly vessel tortuosity) may provide a further simple cue to assist in glaucoma diagnoses and monitoring.

## Supplemental Data

Supplemental Figure 1 Detection of vessel edges (yellow lines) and centerline (blue lines) for a typical retinal image by the automated QUARTZ system. White lines show measures for 1 vessel segment, at low and higher magnification; x,y coordinates and length (pixels) for the vessel segment of interest are shown in the box, along with summary measures for the segment (ie, number of diameters measured, mean (SD) diameter, maximum and minimum diameter, segment length, and a basic measure of tortuosity (vessel length / chord length)).

Supplemental Figure 2 Cross-sectional associations across individuals with same diagnosis in both eyes. Adjusted means (error bars are 95% confidence intervals) of retinal vascular measures by quintiles of corneal compensated intra ocular pressure (IOP_C). Lines represent the line of best fit. Adjusted means are form a multilevel model adjusting for age and sex as fixed effects and random effect for person. Analyses are restricted to individuals with the same diagnosis in both eyes. The coloured symbols and lines are as follows: Black are unaffected individuals (n = 3437); Red are high tension open angle glaucomas (HTG) (n = 32); Grey are normal tension glaucomas (NTG) (n = 26); Green are ocular hypertensives (OHT) (n = 164); Blue are glaucoma suspects (GS) (n = 164). None of the linear trends are formally statistically significant except for: Arteriolar width in unaffected individuals (*P* = .01); venular width in cases of HTG (*P* = .01); venular tortuosity among OHT (*P* = .008) venular area among HTG (*P* = .003).

Supplemental Figure 3 Average between-eye differences in retinal vessel measures by quintiles of between eye differences in corneal compensated IOP among individuals who do not have a glaucoma diagnosis in either eye. R2 values from linear regression using IOP as a continuous variable are less than 0.01 in all instances.

Supplemental Figure 4 Average between-eye differences in retinal vessel measures by quintiles of between-eye differences in Goldmann-correlated IOP among individuals with a different diagnosis between eyes. This is for all different diagnostic pairs combined.

Data Profile
