# Supplementary figures and images for: Retinal Vasculometry Associations With Glaucoma: Findings From the European Prospective Investigation of Cancer–Norfolk Eye Study
Source: Am J Ophthalmol. 2020 Dec;220:140–51. doi: 10.1016/j.ajo.2020.07.027 (PMC7706353; doi:10.1016/j.ajo.2020.07.027)

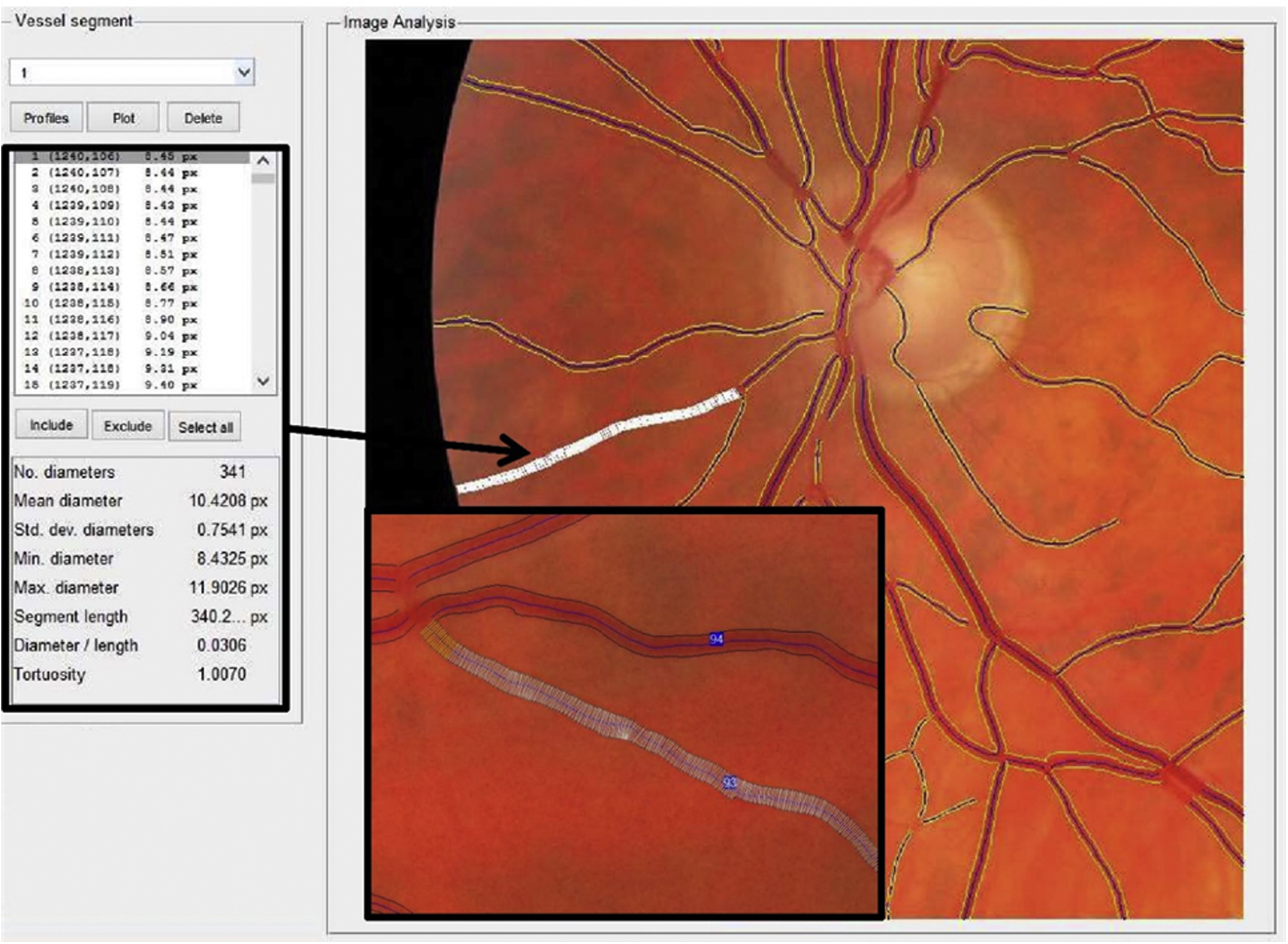

Supplement: Supplemental Figure 1 — Detection of vessel edges (yellow lines) and centerline (blue lines) for a typical retinal image by the automated QUARTZ system. White lines show measures for 1 vessel segment, at low and higher magnification; x,y coordinates and length (pixels) for the vessel segment of interest are shown in the box, along with summary measures for the segment (ie, number of diameters measured, mean (SD) diameter, maximum and minimum diameter, segment length, and a basic measure of tortuosity (vessel length / chord length)). [file figs1.jpg]

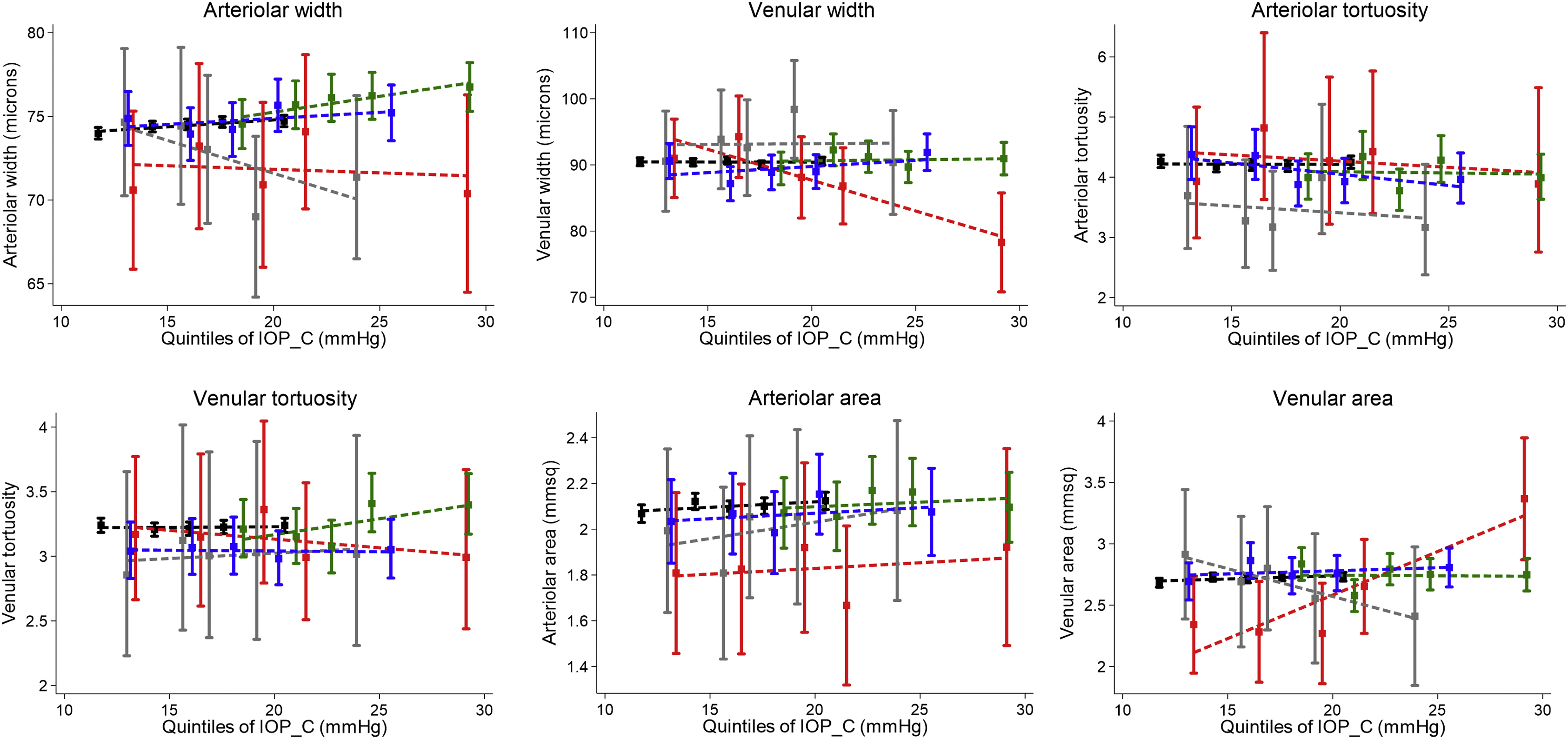

Supplement: Supplemental Figure 2 — Cross-sectional associations across individuals with same diagnosis in both eyes. Adjusted means (error bars are 95% confidence intervals) of retinal vascular measures by quintiles of corneal compensated intra ocular pressure (IOP_C). Lines represent the line of best fit. Adjusted means are form a multilevel model adjusting for age and sex as fixed effects and random effect for person. Analyses are restricted to individuals with the same diagnosis in both eyes. The coloured symbols and lines are as follows: Black are unaffected individuals (n = 3437); Red are high tension open angle glaucomas (HTG) (n = 32); Grey are normal tension glaucomas (NTG) (n = 26); Green are ocular hypertensives (OHT) (n = 164); Blue are glaucoma suspects (GS) (n = 164). None of the linear trends are formally statistically significant except for: Arteriolar width in unaffected individuals (P = .01); venular width in cases of HTG (P = .01); venular tortuosity among OHT (P = .008) venular area among HTG (P = .003). [file figs2.jpg]

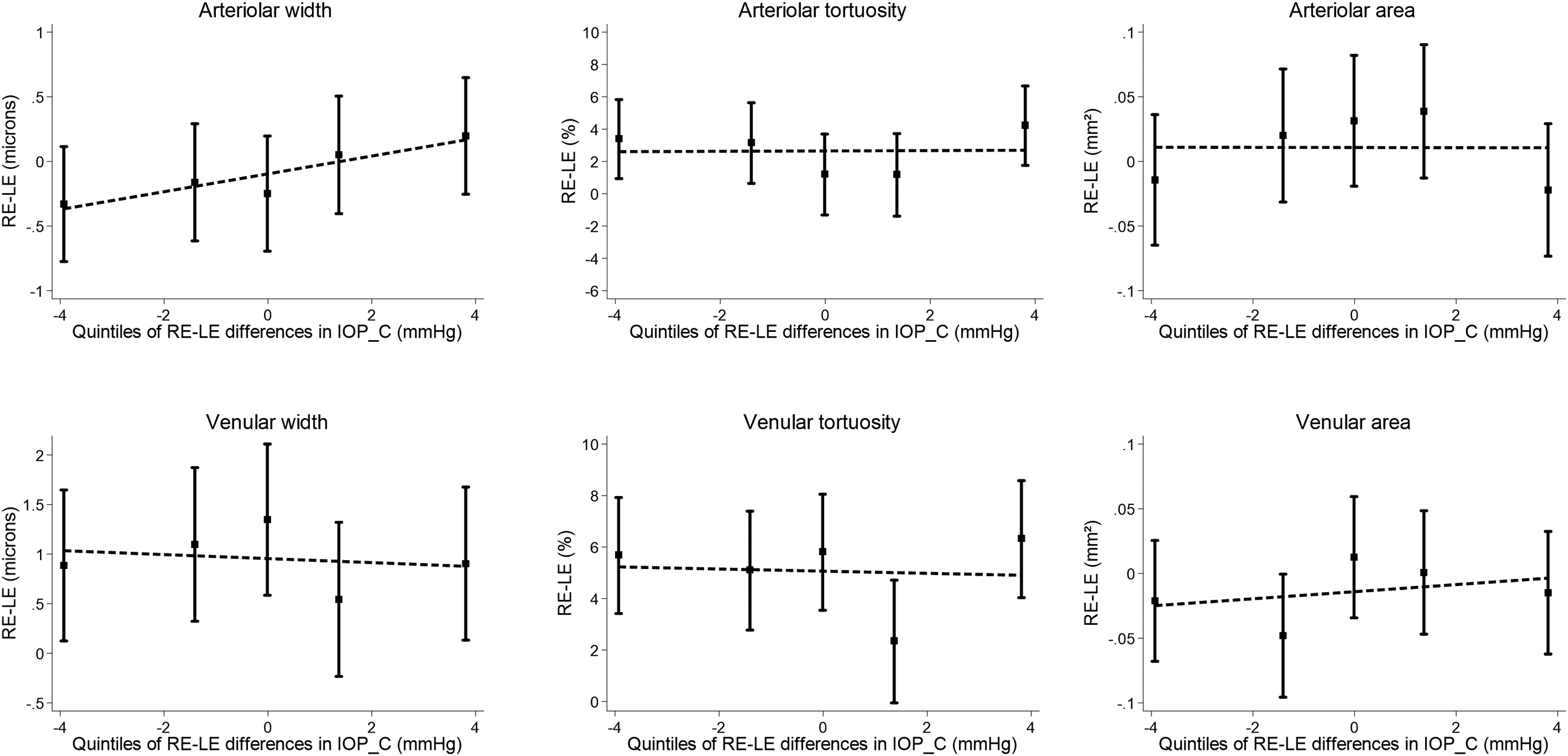

Supplement: Supplemental Figure 3 — Average between-eye differences in retinal vessel measures by quintiles of between eye differences in corneal compensated IOP among individuals who do not have a glaucoma diagnosis in either eye. R2 values from linear regression using IOP as a continuous variable are less than 0.01 in all instances. [file figs3.jpg]

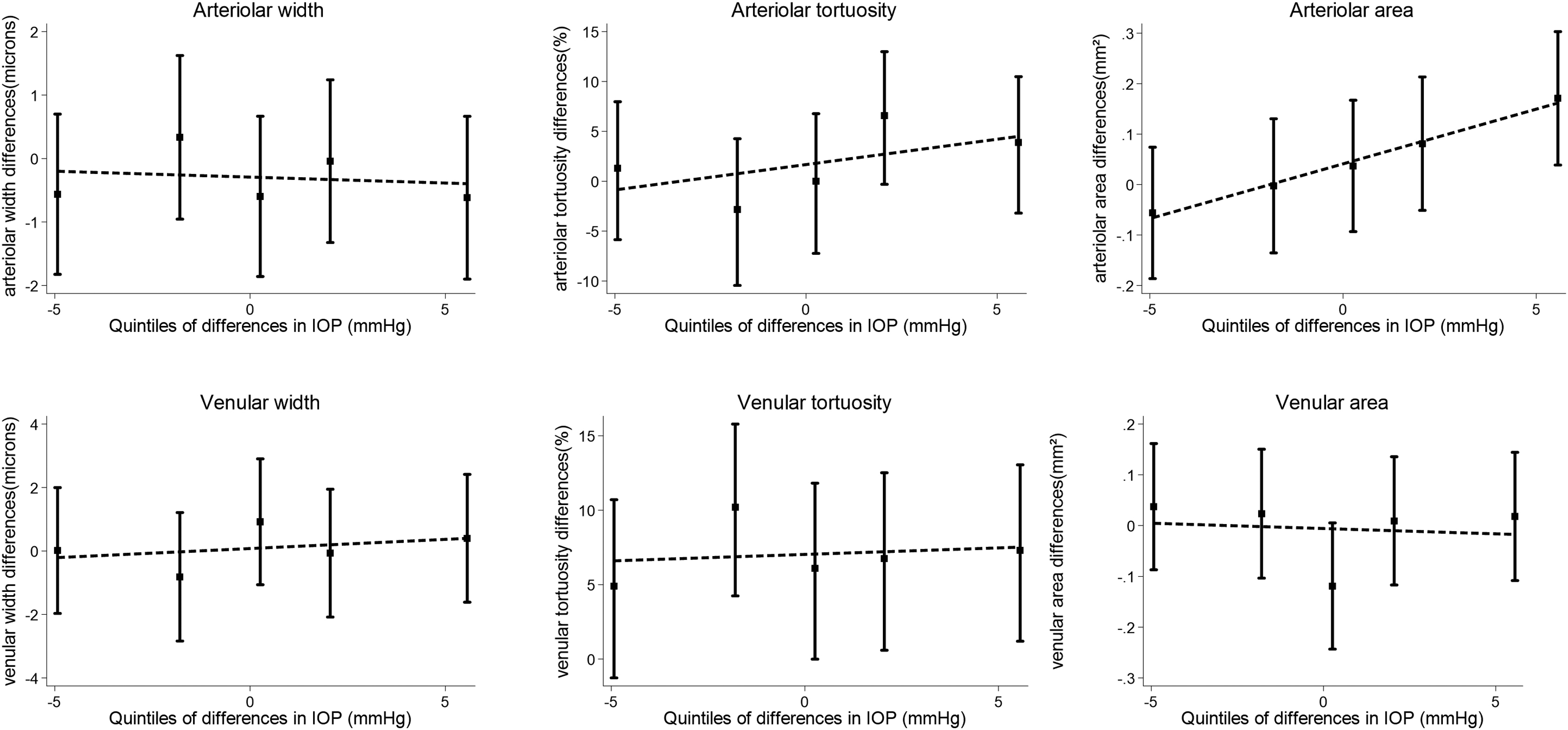

Supplement: Supplemental Figure 4 — Average between-eye differences in retinal vessel measures by quintiles of between-eye differences in Goldmann-correlated IOP among individuals with a different diagnosis between eyes. This is for all different diagnostic pairs combined. [file figs4.jpg]
