# Supplementary material for: Retinal Vasculometry Associations With Glaucoma: Findings From the European Prospective Investigation of Cancer–Norfolk Eye Study
Source: Am J Ophthalmol. 2020 Dec;220:140–51. doi: 10.1016/j.ajo.2020.07.027 (PMC7706353; doi:10.1016/j.ajo.2020.07.027)
Supplement: Supplemental Table [file mmc2.docx]

**Supplemental Table 1: Between eye differences in intra ocular pressure and retinal vascular measures among individuals with a different diagnosis between eyes**

|  |  | **Average between eye differences (95%CI)** | | | | | |  |  |
| --- | --- | --- | --- | --- | --- | --- | --- | --- | --- |
| **Diagnostic** | **N** |  |  | **Vessel width (microns)** | | **Vessel tortuosity (%)** | | **Vessel area (mm^2^)** | |
| **pairs** |  | **IOPg (mmHg)** | **IOPcc (mmHg)** | **Arteriolar** | **Venular** | **Arteriolar** | **Venular** | **Arteriolar** | **Venular** |
|  |  |  |  |  |  |  |  |  |  |
| Unaff vs NTG | 10 | -0.8 (-2.9, 1.2) | -0.1 (-2.2, 2.0) | 1.2 (-2.5, 4.8) | 4.2 (-1.1, 9.4) | 7.8 (31.5, -24.0) | -6.3 (14.4, -32.0) | -0.11 (-0.57, 0.36) | -0.27 (-0.73, 0.19) |
| Unaff vs GS | 106 | -0.5 (-1.1, 0.1) | -0.9 (-1.6, -0.2) | -0.3 (-1.6, 1.0) | 0.3 (-1.7, 2.3) | -6.5 (1.0, -14.5) | 2.1 (8.1, -4.2) | -0.02 (-0.14, 0.10) | -0.06 (-0.19, 0.07) |
| Unaff vs OHT | 288 | -3.0 (-3.3, -2.6) | -3.0 (-3.4, -2.6) | 0.0 (-0.7, 0.7) | -0.4 (-1.6, 0.7) | -1.3 (2.6, -5.2) | -1.6 (2.1, -5.5) | -0.05 (-0.13, 0.02) | 0.00 (-0.07, 0.07) |
| HTG vs GS | 21 | 1.6 (-0.8, 3.9) | 2.0 (-0.3, 4.3) | -4.6 (-8.2, -1.0) | -6.0 (-12.6, 0.7) | 6.4 (18.2, -7.0) | -2.8 (11.2, -19.0) | -0.06 (-0.34, 0.23) | -0.04 (-0.34, 0.26) |
| NTG vs GS | 23 | 0.5 (-0.6, 1.5) | 0.6 (-0.5, 1.8) | 1.3 (-1.0, 3.6) | 2.2 (-1.3, 5.7) | 3.2 (22.0, -20.3) | -14.8 (-0.4, -31.2) | 0.07 (-0.28, 0.42) | -0.19 (-0.48, 0.09) |
| GS vs OHT | 31 | -1.2 (-2.4, 0.1) | -0.6 (-2.1, 0.8) | -0.7 (-2.5, 1.1) | 1.4 (-1.2, 4.0) | -2.1 (8.6, -14.0) | -2.3 (10.3, -16.8) | 0.05 (-0.21, 0.30) | 0.03 (-0.13, 0.19) |

CI = confidence interval, IOPg = Goldmann correlated intra ocular pressure, IOPcc = corneal compensated intra ocular pressure, N= number of participants, Unaff = unaffected
